# Acute and chronic application of novel incremental breath‐hold technique increases endurance exercise performance in healthy men and male military personnel

**DOI:** 10.14814/phy2.71056

**Published:** 2026-08-12

**Authors:** Matthew J. Burley, Benjamin T. Wall, Bert Bond, Craig A. Williams, Alistair J. Monteyne, Francis B. Stephens

**Affiliations:** ^1^ Department of Public Health and Sport Sciences, Faculty of Health and Life Sciences University of Exeter Exeter UK

**Keywords:** apnoea, cardiorespiratory, exercise performance, hematocrit, hemoglobin

## Abstract

We evaluated acute (Study A) and chronic (Study B) effects of a novel breath‐hold technique (BHT) protocol on performance in military personnel. In Study A, 11 healthy male participants completed either rest or a 40‐min BHT before 15 min steady‐state (SS) cycling at 80% V̇O_2max_ and a 5‐min time trial (TT). BHT acutely increased V̇O_2_ (200.3%, *p* = 0.005), produced a significant rise in hemoglobin (Hb; 3%, *p* = 0.030), and altered end‐tidal oxygen (ETO_2_; 2.99%, *p* = 0.054), and lower end‐tidal carbon dioxide (ETCO_2_; 12.1%, *p* = 0.065), reflecting changes in pulmonary gas exchange following breath‐hold exposure, during rest. During SS, blood lactate was lower with BHT (−12.2%, *p* = 0.051; subsample *n* = 3). TT work was greater after BHT (84.4 ± 12.8 kJ vs. 78 ± 13.4 kJ; *p* = 0.019). In Study B, 48 United States Marine Corps Officer Candidates completed 3 weeks of BHT during training, with performance assessed via 3‐mile run time. Run performance improved in both groups (BHT 7.2%; CON 4.9%). Hb concentration increased in BHT (4.3%; *p* = 0.037), but declined in CON (−6.6%). Pre‐test arterial oxygen saturation (S_P_O_2_) was higher in BHT (97.7% ± 0.9% vs. 96.9% ± 1.6%; *p* = 0.03), while estimated partial pressures of arterial oxygen (P_a_O_2_) and carbon dioxide (P_a_CO_2_), used as indices of systemic gas exchange status, were similar between groups. These findings suggest that acute responses to BHT may translate into adaptations when repeated, supporting BHT as an adjunct to endurance training and warranting larger, mechanistic trials.

## Graphical abstract

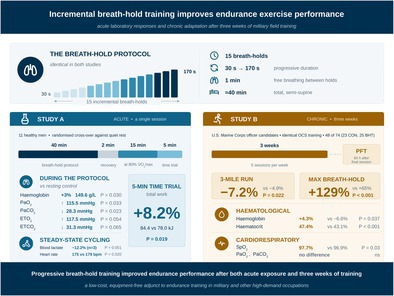

## 1 INTRODUCTION

Optimal physical endurance exercise performance is crucial in athletic disciplines and military operations, and novel training methods are constantly sought after to improve endurance and delay fatigue. Innovative practices such as repeated breath holding have recently gained attention due to the effect on blood gases and increase in circulating hemoglobin (Hb), which appears to be just as potent as known performance enhancing strategies, such as altitude training (Burley et al., 2022; Declercq et al., 2024; Lemaître et al., 2010; Levine & Stray‐Gundersen, 1997; Zoretić et al., 2014) For example, three maximal breath holds interspersed by 2 min of rest led to Hb increases of 2.7% in divers, 2.1% in skiers, and 1.4% in untrained subjects (Richardson et al., 2005), whilst altitude exposure, living high (2500 m) and training low (1250 m), for 4 weeks increased V̇O_2max_ by 5% with 9% higher red‐cell mass and modest (1%–2%) 5000‐m performance gains (Levine & Stray‐Gundersen, 1997), whilst 30‐day blocks at 2000–2200 m also elevate Hb (+1.1–1.4 g/dL) and V̇O_2max_ (+0.9–1.7 mL/kg/min) (Dragos et al., 2022). However, studies of repeated breath holds have generally found no significant improvement in V̇O_2max_ or exercise performance (Citherlet et al., 2021; du Bois, 2014; Sperlich et al., 2015; Yıldız, 2018), perhaps because the cardiorespiratory and hematological effects of a series of breath‐holds typically dissipate in under 10 min (Engan et al., 2013; Espersen et al., 2002; Richardson et al., 2005).

The transient nature of acute breath hold interventions has led to interest in the potential of chronic breath hold training to promote cardiorespiratory and hematological adaptation and improve exercise performance. For example, (Zoretić et al., 2014) demonstrated that 45 min of repeated breath‐holds whilst walking, interspersed by resting periods of three breaths, three times a week for 8 weeks (24 sessions), led to a 5% and 11% increase in Hb and V̇O_2max_, respectively. Whilst exercise performance was not measured, it is suggested that increasing Hb concentration by up to 5% increases V̇O_2max_ by around 10% and endurance exercise performance by up to 40% (Jones & Tunstall Pedoe, 1989). Indeed, following 10 maximal breath holds per day for 28 days, (Son et al., 2020) reported ~3%–4% faster times in the 4th and 5th set of repeated 50 m swim trials, indicating improved fatigue resistance and performance during maximal exercise. In contrast, (Bouten et al., 2022) reported no improvements in Hb mass, V̇O_2peak_, or 3 km cycling time trial performance following 6 weeks of daily statistic apnea training (5 maximal breath holds per session). This suggests that the combined hypoxic–hypercapnic stimulus generated during each breath‐hold session may have been insufficient, or insufficiently sustained, to elicit chronic performance‐related adaptations (Levine & Stray‐Gundersen, 1997; Richardson et al., 2012; Schagatay et al., 2012). Notably, Joulia et al. (2003), who directly measured P_a_O_2_ during breath‐hold training, reported only moderate hypoxia (~65 mmHg at break‐point) and no improvement in V̇O_2max_, although Hb was not measured.

To address this limitation, we have recently demonstrated that a novel method, consisting of progressively increasing repeated breath‐hold duration by 10 s every breath‐hold from 30 s separated by consistent 1 min rest (15 breath‐holds; total breath‐hold duration 25 min), was designed to augment the combined hypoxic‐hypercapnic stimulus and elicited substantial acute perturbations in end‐tidal gases (ETCO_2_≈50 mmHg; ETO_2_≈61 mmHg), alongside increases in Hct (6.4%), Hb concentration (9.2%), ETO_2_ (8.1%), and breath‐hold time during facial submersion (37.4%) in healthy males (Burley et al., 2022). To our knowledge, this was the largest increase in breath‐hold duration recorded in the literature following prior voluntary breath‐holding and the first documented use of these techniques in a laboratory experiment that had measured cardiorespiratory and hematological responses. Whether such an acute hematological response could be reproduced and translate into measurable performance gains in healthy males remained to be tested. Given such notable acute effects of the breath hold method, the aim of the present study was to determine if a single breath hold protocol could improve exercise performance, and if daily breath hold training for 3 weeks could lead to more chronic cardiorespiratory and hematological adaptation, and improve exercise performance. We investigated the effects in elite military personnel to increase ecological validity by enabling chronic breath‐hold training to be evaluated within a highly standardized operational environment, characterized by enforced routines for sleep, diet, and physical training, thereby minimizing behavioral variability between groups (Fallowfield et al., 1998) while preserving real‐world applicability.

## 2 METHODS

### 2.1 Participants

Eleven healthy male participants (mean age: 28.5 ± 6.9 years; height: 178.3 ± 5.2 cm; weight: 84.7 ± 7.1 kg) took part in the first experimental study (Study A) to investigate the acute effect of a single application of the breath‐hold technique (BHT) compared to control (CON) in a randomized cross‐over design. The second experimental study (Study B) was a parallel design investigating the chronic effect of daily application of BHT for 3 weeks, where 74 healthy male USMC participants were recruited and randomized into a CON or BHT group at the Officer Candidate School (OCS). Twenty‐six participants were removed from Officer Candidate School (*n* = 8 CON, *n* = 18 BHT) due to failure to meet the training standards. Consequently, 23 participants were in CON (25.7 ± 2.6 years; 173.6 ± 21.7 cm; 70.3 ± 2.4 kg) and 25 participants were in BHT (25.1 ± 2.8 years; 173.4 ± 20.5 cm; 70.4 ± 3.5 kg). Participants in both studies were non‐smokers and were not suffering from any cardiovascular, neurological, respiratory, or metabolic diseases. Furthermore, none of the participants had been splenectomised and were not taking any prescribed medication.

Prior to participation, all individuals provided written informed consent after receiving a detailed explanation of the experimental protocol and its associated risks. Ethical approval for both studies was obtained from the University of Exeter Sport and Health Sciences Ethics Committee, in accordance with the Declaration of Helsinki (Study A reference: 2054571, Study B reference: 21‐10‐20‐A‐01). Study B was also approved by the U.S. Marine Corps Human Research Protection Official and Chair, Institutional Review Board (IRB), 1019 Elliot Road, Quantico, VA 22134 (approval reference: IRB 00006871). All participants were informed about the nature and risks of the testing procedures and experiment before written informed consent was obtained.

### 2.2 Breath‐hold technique (BHT) protocol

The BHT protocol was the same in both studies and was chosen based on our previous research comparing three breath‐hold techniques (Burley et al., 2022). Each breath‐hold training (BHT) session lasted approximately 40 min and consisted of 15 repeated breath‐holds, performed while participants rested quietly in a semi‐supine position on a bed either in the University of Exeter Nutritional Physiology Research Unit laboratories (Study A), or the participants own bed in their respective Squad Bay at the USMC (Study B), under supervision of the study investigator (M.B.). Participants were asked to gradually extend the duration of repeated breath‐holds from 30 s to 2 min and 50 s, with 1 min of voluntary breathing intervals between each hold. An Iphone app (STAmina, Squarecrowd Apps LLC, Latvia) was used during the BHT, providing verbal timed rest and breath‐hold instructions to the participant. Participants were instructed to perform a ‘breath‐up’ maneuver 10 s before each breath‐hold, which involved taking a deep breath (approximately 80% of their maximal inspiratory volume), exhaling completely, and then inhaling fully before holding their breath. They had the option of using nose clips during the breath‐holds. Regular checks were conducted by the investigator to ensure participants remained alert, conscious, and safe throughout the BHT sessions. The CON condition consisted of 40 min of quite rest whilst semi‐supine. No issues arose during the use of this technique, and all training was performed safely.

### 2.3 Study A experimental protocol

Two weeks before the first experimental visit, participants underwent an incremental cycling test to determine their maximal oxygen consumption (V̇O_2max_) on an electronically braked cycle ergometer (Lode Excalibur Sport, Lode, the Netherlands). Briefly, following a 3 min cycle warm‐up at 20 W, participants commenced cycling at a power output of 100 watts. The resistance was then increased by 35 watts every 3 min until participants reached volitional exhaustion. Participants wore a face mask which was connected to a breath gas analyzer (Metalyzer^®^ 3B, Cortex, Germany) throughout the duration of the test, which measured V̇O_2_ and V̇CO_2_ on a breath‐by‐breath basis. From this test, each participant's individual *α* value (*α* = Power [W]/Cadence [rpm]) was calculated using data from the final completed stage. Additionally, 80% of each participant's V̇O_2max_ was used to determine the target intensity for a steady‐state (SS) cycling bout. Following a 10 min rest, participants were familiarized with a 15 min SS cycle followed by a 5 min cycling time trial (TT) protocol. Bike positions and face mask sizes were individually documented and utilized for all experimental visits.

For each experimental visit participants arrived at the laboratory following a 2 h fast, having abstained from alcohol, caffeine, and strenuous exercise for the prior 24 h, and rested semi‐supine on a bed. A cannula was inserted retrogradely into a dorsal hand vein of the right arm and placed in a heated hand unit (55°C), which was kept patent by a 0.9% sodium chloride drip (~20 mL/h) to allow for subsequent arterialised venous blood sampling. A facemask connected to a breath analyzer (Metalyzer^®^ 3B, Cortex, Germany) and chest heart rate monitor (Polar H10, Polar Electro, Finland) were then fitted, and participants rested quietly for 20 min. Participants continued resting for 40 min (CON) or underwent the 40 min BHT protocol (BHT). Thereafter, participants recovered for 2 min to allow restoration of splenic volume following contraction (Espersen et al., 2002), whilst moving to the ergometer to undergo the SS and TT. Blood samples and respiratory variables (V̇O_2_, V̇CO_2_, FEO_2_, and FECO_2_, exported into 10 s time intervals) were collected before and every 10 min during the rest period and before and every 5 min for the SS and TT phases. There were no differences in laboratory temperature (19.7°C ± 0.8°C vs. 19.4°C ± 0.7°C, *p* = 0.3499) or humidity (46% ± 6% vs. 45% ± 5%, *p* = 0.6767) between CON and BHT trials, respectively, and participants were fan‐cooled whilst cycling.

### 2.4 Study B experimental protocol

Study B experimental protocol was integrated into the standard USMC OCS physical fitness test (PFT) program, which required Candidates to complete an induction, intermediate, and final PFT within their military training program. Participants adhered to a strict OCS dietary regimen that remained consistent across all groups. The induction PFT was performed 2 days before the start of a 3‐week BHT training program conducted five times per week starting at 6 pm each evening, and the intermediate PFT was performed in the morning, 60 h after the final BHT session. The final PFT took place 4 weeks after the Intermediate PFT, following a recovery period. A major part of the PFT required participants to complete a ‘best effort’ 3‐mile run. The run was conducted outdoors on a flat surface in temperatures of −0.6 (induction PFT), −3.3 (intermediate PFT), and 1.1°C (final PFT). All participants began their 3‐mile run together within their respective groups, with run time recorded using a timing gate (Raceclock, Electro‐Numerics, Inc), backed up by a stopwatch (Adanac 3000, Marathon) by OCS physical training instructors. In accordance with standard OCS safety procedures, all participants completed a mandatory water break immediately prior to each PFT. A finger prick blood sample was taken 2 days before and immediately at the finish line, in the standing position, after each run using a safety‐lancet (Normal 21G, Sarstedt, COUNTRY), and subsequently directed to their water station. One day after each PFT in the evening, participants were asked to lay in a semi‐supine position on their beds in their respective squad bays and instructed to fully inhale, exhale, inhale and perform a maximal breath‐hold (MBH). MBH time was recorded using a stopwatch (Adanac 3000, Marathon) for each participant.

### 2.5 Sample collection and analysis

In both studies, whole blood (arterialised‐venous blood in Study A, capillary blood in Study B) was collected into lithium‐heparin tubes (Microvette^®^ CB 300 LH, Sarstedt) and analyzed immediately for Hb concentration using a microcuvette system (HemoCue Hb 201+, HemoCue, Sweden). Due to operational constraints inherent to the military training environment, Hb concentration was obtained as a single immediate capillary measurement post‐exercise in Study B. Hct, partial arterial pressure of oxygen (P_a_O_2_), partial arterial pressure of carbon dioxide (P_a_CO_2_), oxygen saturation (S_p_O_2_), and lactate were analyzed using blood gas analyzers (ABL9, Radiometer, Denmark; Biosen C‐Line Glucose and Lactate Analyzer, EKF Diagnostics, United Kingdom). Unfortunately, Study A experienced technical difficulties resulting in faulty data for Hct, P_a_O_2_ and P_a_CO_2_, and lactate was only measured in three of the 11 participants. As a result, P_a_O_2_ was estimated at all time points using the alveolar gas equation. In Study B, due to logistical constraints related to COVID‐19, blood analyzers were not available to assess baseline samples prior to the Induction PFT.

In Study A, P_a_O_2_ was estimated using a modified form of the alveolar gas equation: P_a_O_2_ = PIO_2_ − (PACO_2_/R) − A–a gradient. Where PIO_2_ is the inspired oxygen partial pressure, calculated as (PB − PH_2_O) × FIO_2_ = 149.7 mmHg at sea level. This takes into account that barometric pressure is 760 mmHg at sea level, water vapor pressure (PH_2_O) is always 47 mmHg at body temperature (37°C), and the fraction of inspired O_2_ is equal to 0.2093 for room air. PACO_2_ was estimated as FECO_2_/R where R is the respiratory exchange ratio (V̇CO_2_/V̇O_2_). The A–a gradient was estimated based on participant age and exercise intensity. At rest, A–a gradient = (Age/4) + 4 was used (McFarlane & Imperiale, 1994). During SS at 80% V̇O_2max_, 20–30 years = 15 mmHg, 31–40 years = 16 mmHg, 41–50 years = 17 mmHg, and during TT, 20–30 years = 25 mmHg, 31–40 years = 26 mmHg, 41–50 years = 27 mmHg was used (Dempsey & Wagner, 1998). Thus, exercise intensity is incorporated by assigning higher (A‐a) O_2_ values at higher intensities (SS vs. TT), with age‐specific constants taken from published exercise physiology data.

### 2.6 Statistical analysis

A paired *t*‐test was employed to detect any differences in total work performed during the TT in Study A, and an independent *t*‐test was used to detect any differences between blood samples analysis taken before the Intermediate PFT in Study B. The assumptions to conduct these parametric statistics were confirmed prior to running all statistics. All other data was assessed using a two‐way analysis of variance (ANOVA) in Study A, and 2‐way ANOVA of MBH and mixed‐effects analysis in Study B for PFT 3‐mile run‐time performance and post‐PFT Hb, with the factors of treatment and time. In cases where a significant main effect was observed, a post‐hoc Sidak test was used with Geisser–Greenhouse correction for sphericity where necessary. Data was analyzed using commercially available software (Prism 9, GraphPad Software Inc., CA, USA). A *p*‐value <0.05 was considered significant. All variables are presented as mean ± standard deviation (SD).

## 3 RESULTS

### 3.1 Study A total work

**FIGURE 1:**
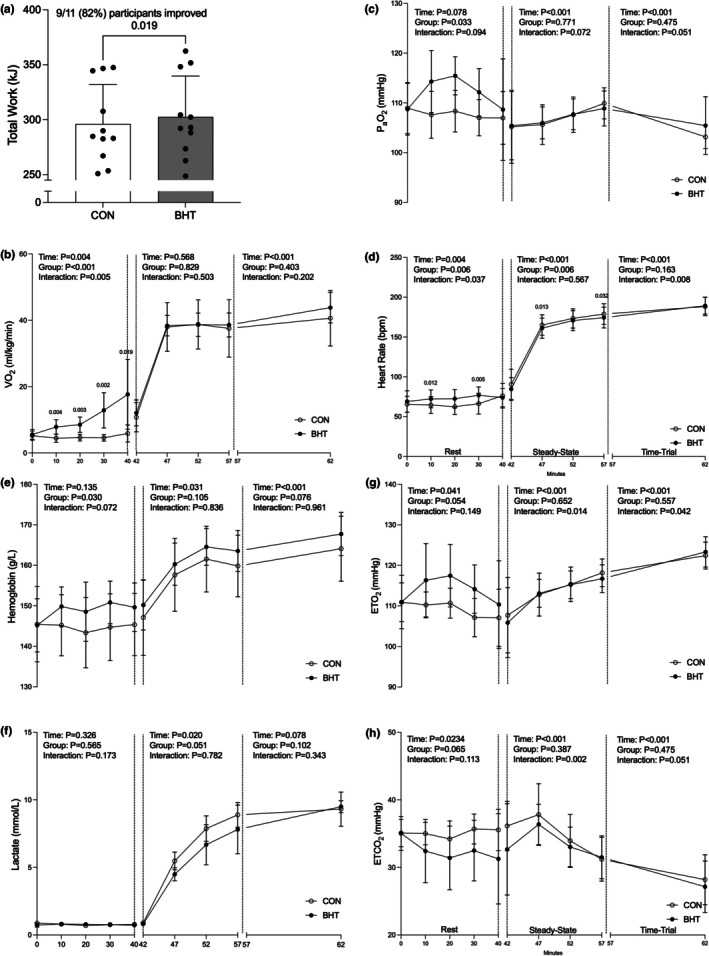
Total work (a), V̇O_2_/kg (b), P_a_O_2_ (c), HR (d), Hb (e), Lactate (f), ETO_2_ (g), and ETCO_2_ (h) response to a 40 min BHT. Blood and cardiorespiratory measurements every 10 min (rest phase), and every 5 min (SS and TT). Total work found using mean load multiplied by time. Main effects of repeated measures ANOVA (b, c, d, e, f, g, h), and a paired *t*‐test (a) presented on each panel. Values are mean ± SD for *n* = 11. *p* < 0.05, *p < 0.001, significantly greater than CON.

Total work performed during the TT (Figure 1a) in BHT was 8.2% greater than CON (84.4 ± 12.8 vs. 78.0 ± 13.4 kJ, respectively; *p* = 0.019).

### 3.2 Study A cardiorespiratory variables

Baseline V̇O_2_ (Figure 1b) was similar between CON (5.2 ± 1.4 mL/kg/min) and BHT (5.5 ± 1.5 mL/kg/min, *p* = 0.986). Thereafter, while V̇O_2_ remained similar after 40 min rest in CON (5.9 ± 2.6 mL/kg/min), it steadily increased throughout BHT to 17.6 ± 10.5 mL/kg/min by 40 min (*p* = 0.005). By the start of SS, V̇O_2_ in BHT had returned to a similar value to CON, with the increase in V̇O_2_ throughout exercise and the subsequent TT the same in BHT and CON. Baseline V̇CO_2_ was similar between CON (4.8 ± 1.5 mL/kg/min) and BHT (5.3 ± 1.8 mL/kg/min, *p* = 0.938). V̇CO_2_ in BHT immediately increased above CON and remained greater throughout rest (*p* = 0.003), reaching 15 ± 7.8 mL/kg/min (*p* = 0.008) after 40 min whilst CON remained similar to baseline (4.9 ± 2.1 mL/kg/min, ns). By the start of SS, V̇CO_2_ was similar in CON and BHT, and increased at a similar rate. During TT, V̇CO_2_ increased in both conditions; however, the increase was greater in BHT (46.6 ± 5.3 mL/kg/min) than CON (41.8 ± 8.8 mL/kg/min, *p* = 0.033).

Baseline P_a_O_2_ (Figure 1c) was similar between CON (109.0 ± 5.1 mmHg) and BHT (108.7 ± 5.2 mmHg, *p* > 0.999). During rest, P_a_O_2_ increased and remained higher in BHT than CON (*p* = 0.033) peaking at 20 min (BHT; 115.5 ± 3.8 mmHg, *p* < 0.001), remaining elevated after 40 min (BHT; 108.7 ± 10.2 mmHg vs. CON; 107.0 ± 5.3 mmHg). During SS, P_a_O_2_ was similar between conditions (CON; 109.9 ± 3.1 mmHg, BHT; 108.9 ± 3.5 mmHg; *p* = 0.072). During TT, P_a_O_2_ decreased in both conditions; however, to a greater extent in CON (103.2 ± 2.3 mmHg) than BHT (105.4 ± 5.8 mmHg; *p* = 0.051). Baseline P_a_CO_2_ was similar between CON (35.1 ± 2.2 mmHg) and BHT (34.5 ± 2.8 mmHg, *p* = 0.983). During rest, P_a_CO_2_ decreased in BHT vs. CON (28.3 ± 7.4 mmHg vs. 35.3 ± 3.3 mmHg, *p* = 0.023). During SS, P_a_CO_2_ decreased in both CON and BHT (27.9 ± 3.5 mmHg vs. 28.0 ± 2.5 mmHg, *p* < 0.001), but P_a_CO_2_ was statistically lower in CON (*p* = 0.005), however, the absolute between‐condition difference was small (~0.1 mmHg) and is unlikely to be physiologically meaningful. During TT, P_a_CO_2_ continued to decrease in both CON and BHT (25.3 ± 4.2 mmHg vs. 23.9 ± 3.4 mmHg, *p* = 0.001), but to a greater extent in BHT (*p* = 0.045).

Baseline HR (Figure 1d) was similar between CON (66 ± 10 bpm) and BHT (69 ± 14 bpm, *p* = 0.918). During rest, HR increased (*p* = 0.037) and remained greater (*p* = 0.006) in BHT than CON (77 ± 10 bpm vs. 66 ± 13 bpm at 30 min, respectively, *p* = 0.005), with values leveling at 40 min (76 ± 15 vs. 74 ± 12 bpm for CON and BHT, respectively, *p* = 0.988). During SS, HR increased in both conditions (CON, 179 ± 13 bpm; BHT, 175 ± 10 bpm; *p* < 0.001); however, HR in BHT was consistently lower (*p* = 0.020). During TT, HR increased in both CON and BHT (188 ± 12 vs. 189 ± 10 bpm for CON and BHT, respectively; *p* < 0.001), but to a greater extent in BHT (*p* = 0.008).

Baseline Hb (Figure 1e) was comparable between CON (145.5 ± 9.3 g/L) and BHT (145.2 ± 6.6 g/L, *p* > 0.999). During rest, Hb was 3% higher than baseline after BHT throughout (149.6 ± 6.0 g/L; *p* = 0.030). In CON, Hb remained similar to baseline (145.4 ± 7.7 g/L). Hb increased during SS (CON, 161.5 ± 8.1 g/L; BHT, 164.5 ± 4.5 g/L) and remained elevated in TT (CON, 164.1 ± 8.0 g/L; BHT, 167.7 ± 5.4 g/L) but with no difference between BHT and CON during these phases. Baseline mean blood lactate concentration (Figure 1f) was similar between CON (0.9 ± 0.0 mmol/L) and BHT (0.7 ± 0.1 mmol/L, *p* = 0.950). In the rest phase, lactate was similar in CON (0.8 ± 0.0 mmol/L) and BHT (0.7 ± 0.0 mmol/L; ns). During SS, lactate rose from baseline in both conditions; mean lactate was lower in BHT than CON (7.8 ± 1.8 mmol/L vs. 8.9 ± 0.9 mmol/L), but this difference did not reach statistical significance (*p* = 0.051). During the TT, lactate remained elevated with no between‐condition difference (CON, 9.3 ± 1.3 mmol/L vs. BHT, 9.5 ± 0.4 mmol/L; *p* = 0.343).

Baseline ETO_2_ (Figure 1g) was similar between CON (110.9 ± 4.8 mmHg) and BHT (111 ± 6.6 mmHg, *p* > 0.999). During rest, ETO_2_ increased and remained higher in BHT compared to CON (*p* = 0.054), peaking at 20 min (BHT; 117.5 ± 7.7 mmHg) and remaining elevated at 40 min (BHT; 110.3 ± 10.8 mmHg vs. CON; 107.1 ± 7 mmHg). During SS, BHT showed lower ETO_2_ (116.7 ± 3.4 mmHg) than CON (118.2 ± 3.4 mmHg, *p* = 0.014), but this reversed during TT, where BHT exhibited higher ETO_2_ (123.3 ± 3.7 mmHg) compared to CON (122.4 ± 3.3 mmHg, *p* = 0.042).

Baseline ETCO_2_ (Figure 1h) was also similar between CON (35.1 ± 2 mmHg) and BHT (35 ± 2.5 mmHg, *p* = >0.999). During rest, ETCO_2_ remained lower in BHT (31.3 ± 6.7 mmHg) than in CON (35.6 ± 2.2 mmHg, *p* = 0.065). In the initial 5 min of SS, ETCO_2_ increased more rapidly in BHT (36.4 ± 3 mmHg) than in CON (37.8 ± 4.6 mmHg), but subsequently declined more sharply in CON (31.2 ± 3.2 mmHg) than in BHT (31.5 ± 3.2 mmHg, *p* = 0.002) over the final 10 min. During TT, ETCO_2_ conitnued to decrease with BHT exhibiting a greater ETCO_2_ reduction (27.1 ± 3.8 mmHg) than CON (28.2 ± 3.7 mmHg, *p* = 0.051). Although this difference approached statistical significance, the absolute between‐condition difference was small (~1.1 mmHg) and should be interpreted cautiously with respect to physiological relevance.

### 3.3 Study B

#### 3.3.1 3‐mile run time

**FIGURE 2:**
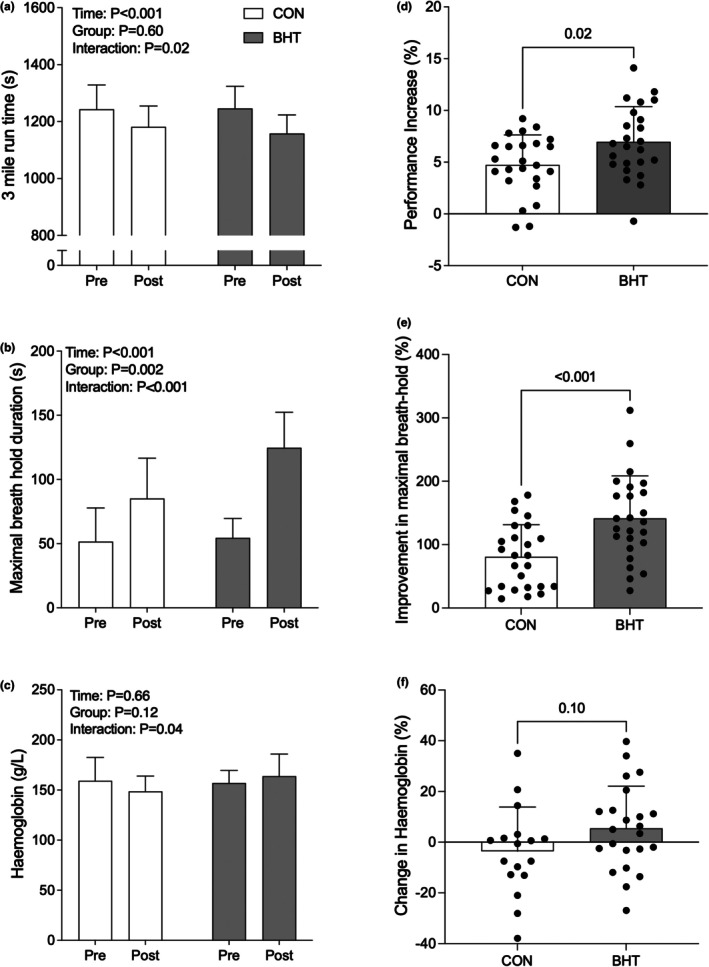
3‐mile run time (a), MBH duration (b), Hb (c), improvement in MBH duration (d), improvement in 3‐mile run time (e), and changes in Hb (f) response to 15 breath‐hold technique sessions over 3 weeks. Blood measurements immediately after the Induction (Pre) and Intermediate (Post) PFT. MBH measurements 1 day after the Induction (Pre) and Intermediate (Post) PFT. Main effects of repeated measures ANOVA (a, b), mixed effects analysis (c), and unpaired *t*‐tests (d, e, f) presented on each panel. Values are mean ± SD for *n* = 48. *p* < 0.05, *p < 0.001, significantly greater than CON.

Induction 3‐mile run time (Pre; Figure 2a,d) was similar between CON (1243.0 ± 85.7 s) and BHT (1247.9 ± 76.5 s; *p* = 0.966). Run time decreased in both groups (*p* < 0.001) following 3 weeks of Officer Candidate training during the Intermediate PFT (Post), but to a greater extent in BHT compared to CON (1158.4 ± 65.1 vs. 1181.6 ± 73.3 s, respectively; *p* < 0.022).

#### 3.3.2 MBH duration recorded 1 day after PFTs

The baseline mean MBH duration was similar between the control (CON) group (51.6 ± 26.2 s) and the breath‐hold technique (BHT) group (54.5 ± 15.1 s), measured 1 day after the Induction Personal Fitness Test (PFT) and prior to any breath‐hold training (Pre, Figure 2b,e). MBH duration increased in both groups (*p* < 0.001) 1 day after the Intermediate PFT, 4 days after the cessation of BHT training (Post) in the experimental group. Furthermore, the BHT group (Post) exhibited a significantly greater increase in MBH duration (124.8 ± 27.7 s) compared to the CON group (85.1 ± 31.4 s; *p* < 0.001).

#### 3.3.3 Hemoglobin measured immediately after PFTs

The baseline mean Hb concentration was similar between the CON group (159.2 ± 23.4 g/L) and the BHT group (157.0 ± 12.6 g/L), measured immediately after the Induction PFT prior to any breath‐hold training (Pre; Figure 2c,f). Immediately after the Intermediate PFT, 3 days after BHT training (Post) in the experimental group, the BHT group exhibited a significantly higher Hb concentration (163.8 ± 22.1 g/L) compared to the CON group (148.7 ± 15.3 g/L; *p* = 0.037).

#### 3.3.4 Hematological response 2 days prior to PFTs

Two days prior to the Intermediate PFT, the Hb concentration was similar (CON, 142.3 ± 12.1 g/L; BHT, 148.2 ± 10.6 g/L; *p* = 0.09), while the Hct was significantly different (CON, 43.1% ± 3.7%; BHT, 47.4% ± 3.4%; *p* < 0.001) between the groups. Two days prior to the Final PFT, both groups exhibited an increase in Hb concentration over time (CON, 159.1 ± 20.6 g/L; BHT, 156.5 ± 22.3 g/L; *p* < 0.001) as well as Hct (CON, 49.7% ± 3.6%; BHT, 50.6% ± 2.9%). However, the CON group had a greater increase in Hb concentration compared to the BHT group (*p* = 0.02), and the BHT group had a greater increase in Hct compared to the CON group (*p* = 0.002). Over the same testing periods, the pH levels remained stable at 7.4 ± 0.0 (*p* = 0.24) in both groups.

#### 3.3.5 Cardiorespiratory response 2 days prior to PFTs

Two days prior to the Intermediate PFT, P_a_O_2_ (CON, 80.2 ± 12.4 mmHg; BHT, 85.0 ± 10.4 mmHg; *p* = 0.18) and P_a_CO_2_ (CON, 38.2 ± 3.9 mmHg; BHT, 38.1 ± 3.1 mmHg; *p* = 0.87) were similar between the groups, while S_P_O_2_ was significantly different (CON, 96.9% ± 1.6%; BHT, 97.7% ± 0.9%; *p* = 0.03). Two days prior to the Final PFT, no significant changes were observed in P_a_O_2_ (CON, 84.2 ± 10.6 mmHg; BHT, 83.3 ± 14.2 mmHg; *p* = 0.13), P_a_CO_2_ (CON, 39.1 ± 3.1 mmHg; BHT, 38.1 ± 3.5 mmHg; *p* = 0.56), or S_P_O_2_ (CON, 97.6% ± 1.1%; BHT, 97.7% ± 1.0%; *p* = 0.09) between the groups.

## 4 DISCUSSION

The primary objective of this study was to evaluate both the acute (Study A) and chronic (Study B) effects of a novel breath hold protocol (BHT) on exercise performance among healthy males and military personnel. Study A demonstrated that BHT administration increased total work output during a 15 min cycling time trial by 8.2% compared to control, which was associated with an increase in Hb concentration and a tendency for an increase in ETO_2_ (proxy for P_a_O_2_). Additionally, 3 weeks of BHT in Study B led to a 7.2% increase in 3‐mile run performance, compared to the 4.9% in control, when performed 60 h after the last BHT, which was also associated with increased hemoglobin concentration, but not P_a_O_2_ or S_P_O_2_. Immediate post‐exercise hemoglobin concentration in Study B may be influenced by exercise‐induced plasma volume shifts rather than proportional changes in hemoglobin mass. Specifically, acute haemoconcentration or hemodilution arising from exercise‐induced plasma‐volume shifts, individual differences in pre‐exercise hydration status and posture, and the use of a single immediate capillary sample per time point in Study B (rather than averaged replicate samples) together contribute to the apparent inter‐individual variability and should be considered when interpreting percentage changes in hemoglobin concentration. The acute effects of BHT appear to accumulate into chronic adaptations when the protocol is repeated. This supports BHT as a low‐cost, portable adjunct to endurance‐training programmes in operational settings, particularly the armed forces, and potentially in other aerobic‐demanding occupations, pending verification in broader cohorts.

The BHT in Study A, which used a 40‐min protocol with 15 breath‐holds each increasing by 10 s in duration and culminating in a final breath hold of 170 s, increased subsequent cycling performance by 8.2% in the healthy male participants. However, most acute studies report no improvement in ‘aerobic’ or ‘anaerobic’ exercise performance (du Bois, 2014; Sperlich et al., 2015; Yıldız, 2018). These studies involved three to five maximal breath holds interspersed by 2‐min rest, however, such protocols reliably evoke splenic contraction and, in some studies, small transient changes in acid–base/respiratory variables, but typically do not alter Hb, Hct, V̇O_2_, or TT outcomes. In Study A, BHT significantly elevated Hb concentration by 3% (*p* = 0.030). Consistent with this, we previously observed increases in Hct (6.4%), Hb concentration (9.2%), and ETO_2_ (8.1%) with the same technique in healthy males (Burley et al., 2022). These data support the view that any performance influence may depend on the magnitude of the hematological response and the underlying hypoxic/hypercapnic load. For example, the present BHT progressed to a supramaximal breath hold duration (Burley et al., 2022), whereas both (Yıldız, 2018) study and (du Bois, 2014) required breath‐holds at least 90% of a pre‐determined MBH, influencing limited progression, and the ‘Wim Hof Method’ performed three cycles of 30 hyperventilation breaths followed by a maximal low‐lung volume breath‐hold (Citherlet et al., 2021). Indeed, in a facial‐immersion protocol of five maximal breath holds (MBHs), (Bourdas, 2021) improved time‐to‐exhaustion by ~11% (49.2 vs. 44.8 s). The authors attributed this to a priming effect, pre‐exercise lower pH, higher bicarbonate, higher ventilation, and higher V̇O_2_, indicating a greater aerobic contribution from the outset, with no change in Hb or Hct. Although not formally measured, participants in Study A frequently reported that endurance exercise during the steady‐state period felt easier following BHT, which aligns with the observed reduction in blood lactate concentration and P_a_CO_2_ in the face of similar P_a_O_2_. This may reflect greater tolerance to arterial oxygen desaturation or altered oxygen utilization efficiency, rather than enhanced convective oxygen delivery, consistent with the absence of changes in VO_2_ kinetics. In addition, the observed acute increase in hemoglobin concentration may enhance hydrogen ion buffering capacity during high‐intensity exercise, potentially contributing to improved tolerance to metabolic acidosis despite the absence of changes in VO_2_ kinetics. However, a psychological component, such as altered perception of effort or increased confidence, cannot be discounted and may also have contributed to this perceived ease.

Study B was intentionally designed as a field‐based investigation to examine the operational relevance of breath‐hold training effects under real‐world military conditions, whereas Study A employed controlled laboratory protocols to enable detailed physiological assessment under exercise intensities and durations comparable to the field task. Study B used BHT five times per week, for 3 weeks, increasing 3‐mile run time performance by 7.2% and MBH duration by 129%. In contrast, most studies implementing breath hold training over 2 weeks–3 months did not report exercise performance improvements (Bouten et al., 2019, 2022; Engan et al., 2013; Joulia et al., 2003). Bouten et al.'s (2019: First four breath holds 80% MBH with 2‐min rest intervals; 2022: Five MBHs with 30 s rest intervals) found no change in Hbmass, V̇O_2peak_, or 3 km TT. Consistent with this dose–response, our earlier increasing breath‐hold (IBH; Burley et al., 2022) increased breath‐hold duration without elevating Hb or Hct, likely reflecting a stimulus characterized by moderate hypoxia (ETO_2_≈82 mmHg) but minimal hypercapnia (ETCO_2_≈38 mmHg), a pattern previously shown to be insufficient to elicit acute hemoglobin increases during breath‐holding (Richardson, 2008; Richardson et al., 2012). Similarly, (Joulia et al., 2003) used short static and dynamic apneas during low intensity cycling over 3 months, while (Engan et al., 2013) employed five MBHs twice daily for 2 weeks. Both studies reported earlier and more pronounced bradycardia, but no increase in exercise performance, or Hb, respectively. In contrast, (Zoretić et al., 2014), who maintained ETCO_2_ above 45 mmHg during repeated breath‐holds while walking at 60% HR_max_, increased Hb by 5% alongside improved V̇O_2max_ by 11%, which is entirely in line with the 4.3% increase in Hb concentration in the present study observed 60 h after the last BHT session. This pattern is reinforced by the acute Hb rise observed in Study A, which was absent in protocols using shorter or non‐progressive breath holds, supporting the view that the magnitude and sustained nature of the hypoxic‐hypercapnic load is a key determinant of the hematological response. Importantly, the control group also improved performance (4.9%) despite a 6.6% fall in Hb, a pattern consistent with training‐induced plasma‐volume expansion, where total blood volume rises but the plasma increase slightly exceeds red‐cell expansion, lowering Hct/Hb while enhancing stroke volume and thermoregulatory capacity, adaptations that can support performance gains even with a small reduction in arterial O_2_ content (Seiler, 2012). Indeed, unlike (Richardson et al., 2005) who found that acute Hb increases returned to baseline within 10 min after the final apnea, both our findings and those of (Woorons et al., 2024) suggest that performance benefits from chronic BHT may persist for up to 5 days but return to baseline within 12 days, even with continued physical activity. This highlights a distinction between the rapid loss of acute physiological effects and the slightly longer retention of performance adaptations.

Trained divers consistently outperform untrained individuals in maximal breath‐hold duration and often exhibit higher circulating Hb (Elia et al., 2021; Richardson et al., 2005; Schagatay et al., 2012), adaptations commonly attributed to repeated exposure to hypoxic‐hypercapnic stress (Levine & Stray‐Gundersen, 1997; Richardson et al., 2012). Prior work has demonstrated that breath‐holding can acutely increase circulating Hb, an effect frequently associated in the literature with splenic contraction during breath‐holding or exercise (Laub et al., 1993; Schagatay et al., 2020; Shephard, 2016). In addition, repeated hypoxic exposure has been shown to stimulate erythropoietin (EPO)‐mediated erythropoiesis, contributing to longer‐term hematological adaptations (Eckardt et al., 1989; Engan et al., 2013; Jelkmann, 2011; Richardson, 2008). While these mechanisms may contribute to breath‐hold‐related adaptations reported previously, spleen volume, splenic contraction, and EPO were not measured in the present study and therefore cannot be directly inferred. Consequently, any reference to splenic involvement should be considered speculative and based on established literature rather than direct evidence. Future studies incorporating direct assessment of splenic responses alongside gas exchange and hematological measures are required to better delineate the relative contribution of acute and chronic mechanisms.

In conclusion, a novel breath‐hold technique, applied both acutely and over 3‐weeks, significantly enhanced exercise performance in elite military personel, as evidenced by improved work output, 3‐mile run times, maximal breath‐hold duration, reduced lactate production (*n* = 3), and increased hemoglobin concentration acutely (Study A) and chronically (Study B). The breath‐hold technique offers a cost‐effective and accessible training method that can enhance both immediate and long‐term physical performance, indicating potential utility for military and other high‐demand occupations. Nevertheless, dose–response guidelines and mechanistic studies (e.g., spleen imaging, EPO/Hbmass) are needed to optimize protocols and establish the durability and scope of adaptations.

## AUTHOR CONTRIBUTIONS

**Matthew J. Burley:** Conceptualization; data curation; formal analysis; funding acquisition; investigation; methodology; project administration; resources; software; visualization. **Benjamin T. Wall:** Validation. **Bert Bond:** Supervision; validation. **Craig A. Williams:** Supervision; validation. **Alistair J. Monteyne:** Project administration; validation. **Francis B. Stephens:** Conceptualization; data curation; formal analysis; investigation; methodology; project administration; supervision; validation; visualization.

## FUNDING INFORMATION

This research was funded by the University of Exeter. M.J.B. received a Postgraduate Education Scheme training grant from the Royal Navy (JSP 750) covering postgraduate tuition only. The Royal Navy had no role in study design, analysis, interpretation, or the decision to publish. Study B was conducted within the U.S. Marine Corps Officer Candidate School training programme under an existing institutional research access arrangement; the U.S. Marine Corps had no role in study design, analysis, interpretation, or the decision to publish.

## CONFLICT OF INTEREST STATEMENT

M.J.B. is the sole director of Burley Performance Labs Ltd. (Companies House No. 16700340), which trades as Adapnia (UK trade mark UK00004268291). A UK patent application (GB Application No. 2616190.1, filed 13 July 2026, “SYSTEMS AND METHODS FOR ADAPTIVE BREATH‐HOLD PROCEDURES”) has been filed by Burley Performance Labs Ltd. covering a computer‐implemented method that adapts breath‐hold procedures for application use; M.J.B. is the named inventor. The remaining authors (F.B.S., A.J.M., B.B., C.A.W., B.T.W.) declare no competing financial or non‐financial interests. None of the authors holds any consulting, advisory, or employment relationship with any company whose products are referenced in this manuscript. The U.S. Marine Corps Officer Candidate School (Quantico, Virginia, USA) provided access to participants and venue support for Study B but had no role in study design, data collection, analysis, interpretation, or the decision to publish.

## ETHICS STATEMENT

Both studies were conducted in accordance with the Declaration of Helsinki and were approved by the University of Exeter Sport and Health Sciences Ethics Committee (Study A reference 2054571; Study B reference 21‐10‐20‐A‐01). Study B was additionally approved by the U.S. Marine Corps Human Research Protection Official and Chair, Institutional Review Board, 1019 Elliot Road, Quantico, VA 22134, USA (approval reference IRB 00006871). All participants received a detailed explanation of the experimental protocol and its associated risks, and provided written informed consent prior to participation.

## Data Availability

Data underlying this study involve military trainees and are subject to confidentiality, GDPR, and governance requirements. De‐identified summary data may be available from the corresponding author on reasonable request, subject to a data‐sharing agreement and required institutional/USMC approvals. No public repository deposition is possible at this time.
